# The Utility of Rectal Water Contrast Transvaginal Ultrasound for Assessment of Deep Bowel Endometriosis

**DOI:** 10.3390/life13051151

**Published:** 2023-05-10

**Authors:** Martyna Masternak, Malwina Grabczak, Tomasz Szaflik, Beata Mroczkowska, Łukasz Mokros, Beata Smolarz, Hanna Romanowicz, Krzysztof Szyłło

**Affiliations:** 1 Department of Gynecology, Operative Gynecology and Treatment of Endometriosis, Polish Mother’s Memorial Hospital Research Institute, Rzgowska 281/289, 93-338 Lodz, Poland; 2 Second Department of Psychiatry, Institute of Psychiatry and Neurology, Sobieskiego 9, 02-957 Warsaw, Poland; 3 Laboratory of Cancer Genetics, Department of Pathology, Polish Mother’s Memorial Hospital Research Institute, Rzgowska 281/289, 93-338 Lodz, Poland

**Keywords:** RWC-TVS, DIE, #Enzian classification, endometriosis

## Abstract

Deep infiltrating endometriosis (DIE) is characterized by the presence of endometrial tissue outside the uterine cavity that infiltrates at least 5-mm deep below the peritoneal layer. Imagining examinations are the first-choice methods to detect DIE. The aim of this study is to assess whether rectal water contrast transvaginal sonography (RWC-TVS) can be a useful tool for the estimation of the size of deep bowel endometriotic nodules. This retrospective study includes 31 patients subjected to RWC-TVS who underwent surgery due to deep bowel endometriosis between January 2021 and December 2022. Nodule dimensions measured via ultrasound were compared to those of histopathological samples taken after surgery. In total, 52% of patients had endometriosis limited only to the intestines, 19% had endometriotic nodules located at uterosacral ligaments and posterior vaginal fornix, 6% at the anterior compartment, and 13% at a different location. Additionally, 6% of patients had nodules at more than two locations. In all but one case, the intestinal nodules could be seen on RWC-TVS images. The largest nodule dimension measured via RWC-TVS and the size of the equivalent histopathological sample correlated (R = 0.406, *p* = 0.03). Thus, RWC-TVS allows for the detection of DIE and moderate estimation of the nodule sizes and should be practiced during a diagnostic process.

## 1. Introduction

Endometriosis is a common inflammatory and estrogen-dependent gynecologic disease defined as the presence of endometrium-like tissue outside the uterine cavity [1]. Endometriosis occurs in about 10% of women of reproductive age, 80% of women with severe pelvic pain, and 20% to 50% of women suffering from infertility [2,3]. The likelihood of developing endometriosis increases after puberty due to elevated estrogen levels, menstruation, sexual activity, and changes in the pelvic microbiome. The frequency of endometriosis decreases progressively thereafter. After the age of 30, the risk of developing endometriosis is rather low [4].

### 1.1. Pathophysiology

The pathophysiology of endometriosis remains unknown, but many theories have been reported [1]. The most common theories postulate retrograde menstruation, embryonic Mullerian rests, coelomic metaplasia, lymphovascular spread, and immune dysregulation [1,5]. However, the pathogenesis and etiology of endometriosis remain unknown [6].

The most popular theory describes retrograde menstruation as a possible cause of the disease [7]. Physiologically, the top layer of the endometrium is shed during menstruation, preparing the endometrium lining for the next menstrual cycle, resulting in vaginal bleeding that lasts an average of five days [8]. During retrograde menstruation, shed endometrial cells migrate through the fallopian tubes into the abdominal cavity and adhere to tissues at ectopic locations [7]. Typical manifestation points of endometriotic lesions supported by this theory are the peritoneum of the fossa ovarica, Douglas cavity, and sacrouterine ligaments [7]. Additionally, the presence of peritoneal fluid in the pelvic cavity presumably contributes to endometriosis development caused by retrograde menstruation [7]. Peritoneal fluid, naturally present in the abdominal cavity in small amounts, is made to lubricate the surface of tissue lining the abdominal wall and pelvic cavity and covers most of the organs at these locations [9,10]. Primarily, among others, produced by the developing follicles and corpus luteum, the peritoneal fluid contains electrolytes; urea; and ovarian steroidal hormones; as well as cellular components, including, for e.g., endometrial cells; macrophages; lymphocytes; and red blood cells [3]. Endometrial cells present in the peritoneal fluid might accumulate in various locations within the pelvic cavity, implant into the lining tissue, and grow [3]. Endometriotic lesions may be superficial but, in many cases, they can penetrate more than 5-mm deep into the tissue, resulting in deep infiltrating endometriosis (DIE) [7]. Interestingly, the volume of peritoneal fluid is significantly higher in women with endometriosis [3].

Retrograde menstruation is a common phenomenon that usually does not cause the pathological phenotype in women, and only about 10% to 15% of women of reproductive age will develop endometriosis caused by retrograde menstruation [7]. This suggests that other factors, such as the type of cells translocated into the peritoneal cavity, genetic and epigenetic processes, environmental influences, or immune diseases, are also important risk factors for the development of endometriosis [7]. Immune cells from patients who suffer from endometriosis secrete proinflammatory and blood vessel growth-promoting factors such as chemokines, cytokines, and metalloproteinases that can additionally contribute to disease pathophysiology and cause persistent inflammation, which causes fibrosis and the formation of adhesions [11,12].

### 1.2. Localization

We can distinguish superficial endometriosis and deep infiltrating endometriosis, the latter being a more severe clinical form that is characterized as an endometriotic lesion infiltrating the peritoneum and penetrating the retroperitoneal space or the wall of the pelvic organs to a depth of at least 5 mm [13]. About 15% to 30% of endometriosis patients experience it [14]. Based on anatomical locations, we can identify endometriosis of the ovary and uterus, anterior compartment (urinary bladder, uterovesical region, and ureters), and posterior compartment (uterosacral ligaments, posterior vaginal fornix, anterior rectum or anterior rectosigmoid junction and sigmoid colon) [15].

### 1.3. Symptoms

The main symptoms of endometriosis are long-term acyclic pelvic pain, dysmenorrhea, deep dyspareunia, dyschezia, dysuria, and infertility. Less frequent symptoms, which can be present especially in deep infiltrating endometriosis, are cyclic bowel and urinary symptoms such as painful bowel movements, abdominal bloating, cyclical diarrhea or constipation, mucus in the stool, rectal bleeding, polyuria, urine urge, and presence of blood in the urine. Symptoms can become worse during menstruation [16,17].

### 1.4. DIE and Infertility

Women with deep infiltrating endometriosis often experience infertility—spontaneous fertility rate ranges from 2 to 10%. Infertility is mainly caused by impaired gamete migration and tubal function due to anatomy changes, adhesion, and a chronic inflammatory environment. Adenomyosis often occurring together with DIE reduces the likelihood of conception. According to the ESHRE guideline from 2022, surgical treatment may be an option in symptomatic patients. The total pregnancy rate after surgical treatment reaches 80% [18,19].

### 1.5. Endometriosis as a Risk Factor

Endometriosis increases the risk of endometrial and ovarian cancer, cardiovascular diseases, autoimmune diseases, and allergies [3,20,21,22]. Epithelial carcinomas of ovaries such as ovarian clear cell carcinoma (OCCC) and endometrioid carcinoma (EnOC) can develop on the grounds of endometriosis. Some gene mutations associated with the occurrence of ovarian cancer directly related to endometriosis have been confirmed, such as somatic cancer driver mutations in the ARID1A gene that were found in a group of patients with endometriosis and correlated with the pathogenesis of ovarian cancers in this subset. As recently reported for estrogen receptor-positive breast cancer, the role of mutations in the ARID1A gene leading to a modulation of estrogen receptor signaling can contribute to the pathogenesis of endometriosis [23,24,25].

### 1.6. Diagnosis

Unfortunately, the diagnostic tools available today are insufficient to confidently diagnose or, at the very least, exclude endometriosis. Only half of the big endometriotic lesions are discovered during a clinical examination [4]. Imagining examinations, e.g., ultrasonography or magnetic resonance imaging (MRI) scans, are methods of choice in diagnosing superficial and deep infiltrating endometriosis.

### 1.7. Treatment

The most popular treatment options include nonsteroidal anti-inflammatory drugs, hormonal contraceptives, progestogens, antiprogestogens, gonadotropin-releasing hormone (GnRH) agonists, and antagonists [3]. Studies currently recommend using assisted reproductive technology (ART) for patients whose endometriosis has caused infertility [3].

When empirical treatment fails, exploratory laparoscopy is recommended. Endometrial tumors may be destroyed, cut, or enucleated during the surgery (laparoscopy or laparotomy) [3,4]. Preoperative evaluation is essential since it is critical to inform the patient about the planned scope of the procedure so they may provide their informed consent for the treatment [14]. A confident evaluation may also help to choose the institution in which the operation can be performed, a specially trained surgical team, the best operation method, and to assess the expected complexity of the operation [26].

### 1.8. Classification

An accurate and general classification is essential to evaluate the disease’s stage, the location of endometriotic changes, and the clinicopathological consequences that the disease may have. To design the best course of treatment, it is crucial to accurately determine the stage of the disease. It allows for the implementation of tailored and targeted treatment that is the best therapeutic alternative for the patient. There are a few classifications of endometriosis to assess the severity of the condition. The American Society for Reproductive Medicine (ASRM) score is the most widely used; however, it ignores the involvement of retroperitoneal structures with deeply infiltrating endometriosis [27]. Contrarily, the Enzian classification includes a morphological description of deep infiltrating endometriosis and correlates with symptoms of the disease [15,28].

**Figure 1:**
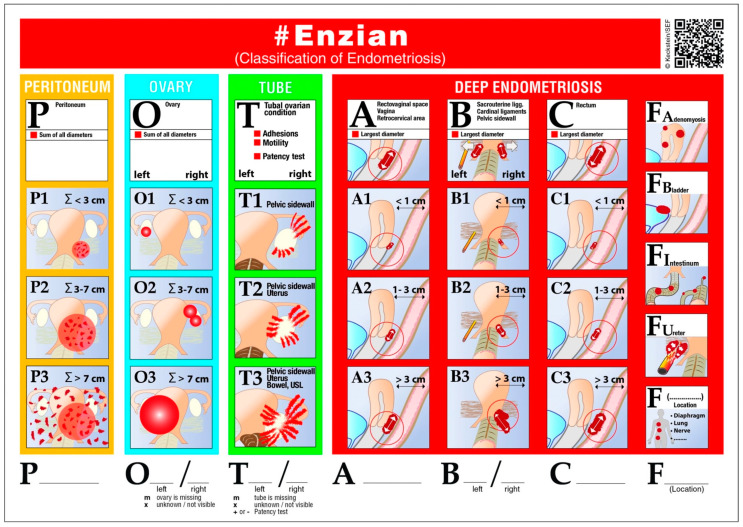
#Enzian classification—picture of affected organs and compartments (https://www.endometriose-sef.de/wp-content/uploads/2021/02/Figure1new-scaled.jpg, accessed on 23 April 2023) [15].

The #Enzian classification, the most current version of the Enzian classification, describes the localization of deep infiltrating endometriosis in a variety of compartments; A for the vagina, rectovaginal space, and rectocervical area; B for the sacrouterine ligaments, cardinal ligaments, and pelvic sidewalls; C for the rectum; and F for the uterine and other extragenital sites, such as FA for the adenomyosis, FB for the urinary bladder, FI for the intestines—particularly the cranial to the rectosigmoid junction, which is about 16 cm above the anal verge, upper sigmoid, transverse colon, cecum, appendix, and small bowel—and finally FU for the ureter and different, far locations [15,28]. Other compartments, such as P for the peritoneum, O for the ovaries, and T for tuboovarian conditions such as adhesions, motility, and tubal patency are included in the improved #Enzian classification [15]. Figure 1 shows the #Enzian classification [15]. It is common practice to categorize endometrial changes according to the #Enzian classification at various stages of therapy. It can be used to assess endometrial changes after surgery or during imaging procedures such as transvaginal ultrasonography and magnetic resonance imaging. Gynecologists, sonographers, radiologists, and surgeons find it useful in their daily practice [15].

### 1.9. Rectal Water Contrast Transvaginal Sonography

Transvaginal sonography (TVS) is the primary imaging method used to diagnose endometriosis due to its high accessibility and low cost [29]. Even though deep intestinal endometriosis prediction accuracy with ultrasound is still a subject of research, the technology has improved over the last few years. Rectal water contrast transvaginal ultrasonography (RWC-TVS) is a result of an attempt at such improvements [14]. To acquire a clearer visualization of the intestinal walls, 300–500 mL of saline solution must be added into the lumen of the rectum [30]. Compartment C from the #Enzian classification is the region of focus during the RWC-TVS assessment [14,15,17]. Compartment C is defined as a distance located up to 16 cm from the anal verge. The intensity grade of endometrial changes is determined by the maximum tumor diameter measured in the sagittal section along the axis of the rectum. Endometrial lesions can be categorized as C1 for changes with a maximal diameter of less than 1 cm, C2 for lesions with a diameter between 1 and 3 cm, and C3 for lesions more than 3 cm [15].

The aim of this study is to evaluate the precision of rectal water contrast transvaginal ultrasound application in estimating the size of nodules in deep bowel endometriosis in routine department practice.

## 2. Material and Methods

### 2.1. Source Data

In this retrospective study/case series, all information about patients was collected from their medical history. The study group consisted of 31 women, who underwent surgery for deep infiltrating endometriosis between January 2021 and December 2022 in the Department of Gynecology, Operative Gynecology and Treatment of Endometriosis at Polish Mother’s Memorial Hospital, Research Institute, Lodz, Poland. Patients with endometriosis that was exclusively present in the reproductive organs were excluded, as were those in whom the pre-operative ultrasound examination was skipped, or the size of the nodules was not measured. The mean age of the study group was 37 years old (ranging from 27 to 49 years old). All women were pre-menopause.

### 2.2. Ultrasound Measurements

Ultrasound procedure and evaluation were carried out in accordance with the recommendations of the International Deep Endometriosis Analysis (IDEA) group by gynecologists with experience in ultrasound examination. All examinations were performed using Voluson GE S10 and E10. In case of suspicion of bowel lesions, rectal water contrast transvaginal ultrasound was performed. RWC-TVS requires injection of 300–500 mL of saline solution through a catheter into the rectum. There are no patient preparation guidelines for this examination. This procedure allows for evaluation of the location of the nodules, the level of invasion into the intestinal wall, and the stenosis degree of the bowel lumen. Dimensions of nodules measured during the ultrasound were compared with the measurement of histopathological samples obtained after surgery. The greatest measured nodule dimensions were considered. The #Enzian classification was used to describe the stage of deep infiltrating endometriosis.

### 2.3. Acquisition and Measurements of Histopathological Samples

Histopathological postoperative samples were measured and examined in the Department of Pathology at Polish Mother’s Memorial Hospital, Research Institute, Lodz, Poland. In our study we retrospectively used and compared results from descriptions of histopathological examinations.

### 2.4. Statistical Analysis

The Shapiro–Wilk test was used to assess the distribution of the data. Wilcoxon paired test and Spearman correlation quotient were utilized to analyze the data. A *p*-value < 0.05 was regarded to indicate statistical significance. Statistical analysis was performed using STATISTICA software [31].

## 3. Results

The RWC-TVS examination showed that 52% of patients had endometriosis limited to the intestines. Meanwhile, 19% had endometriosis nodules at uterosacral ligaments and posterior vaginal fornix, 6% at the anterior compartment (urinary bladder, uterovesical region, and ureters), and 13% at a different location. Moreover, 6% of examined patients had nodules at more than two locations. Table 1 reports the incidence of endometriosis at the aforementioned locations.

The most frequent stages of endometriosis advancement according to the #Enzian classification were P0, T0, O0, A0, B0, and C2 (located in the rectum and sigmoid colon, size of lesions 1–3 cm); and P0, T0, O0, A0, B0, C3 (located in the rectum and sigmoid colon, size of lesions > 3 cm).

**Figure 2:**
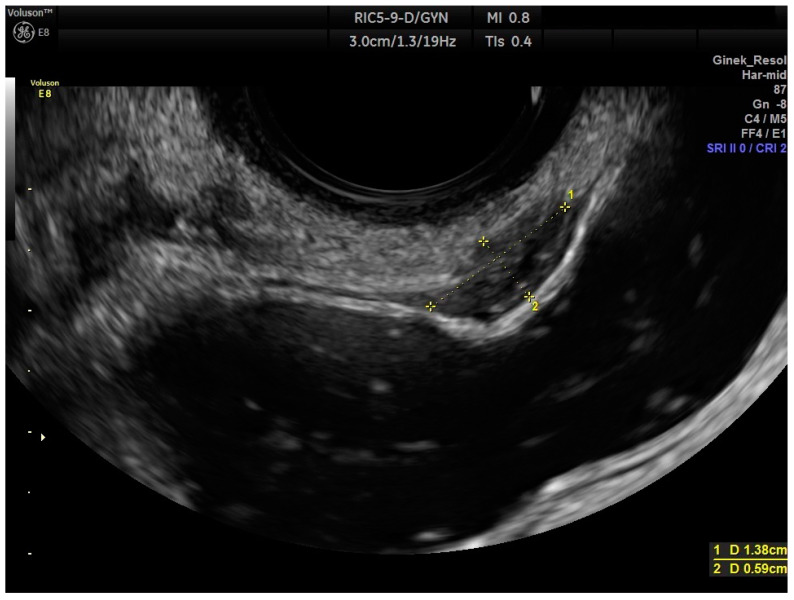
RWC-TVS ultrasonography photograph showing endometrial lesion in the anterior wall of the rectum. Measurements of lesions are shown in yellow dashed lines. (1) Measurement of the length of the lesion, (2) measurement of the width of the lesion.

**Figure 3:**
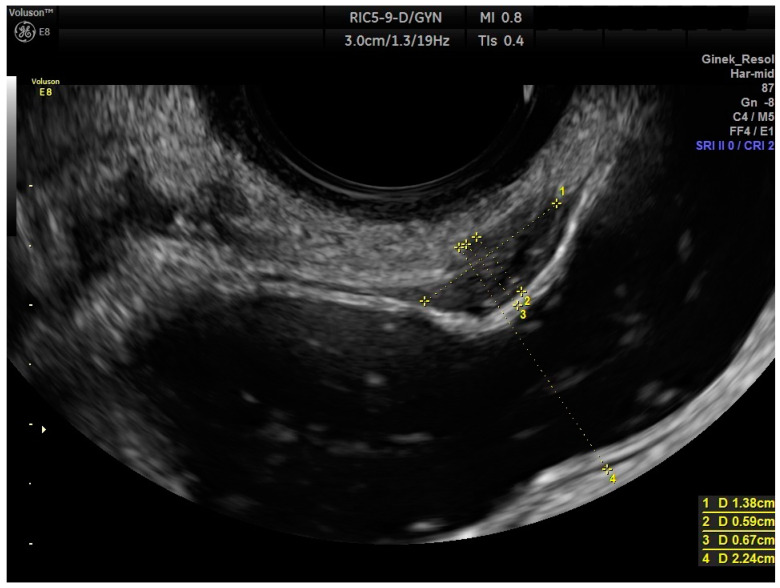
RWC-TVS photograph showing endometrial tumor in the anterior wall of the rectum (bowel occlusion at 30%). Measurements of lesions and bowel occlusion are shown in yellow dashed lines. (1) Measurement of the length of the lesion, (2) measurement of the width of the lesion, (3) measurement of occlusion of the bowel lumen, (4) measurement of the bowel lumen.

**Figure 4:**
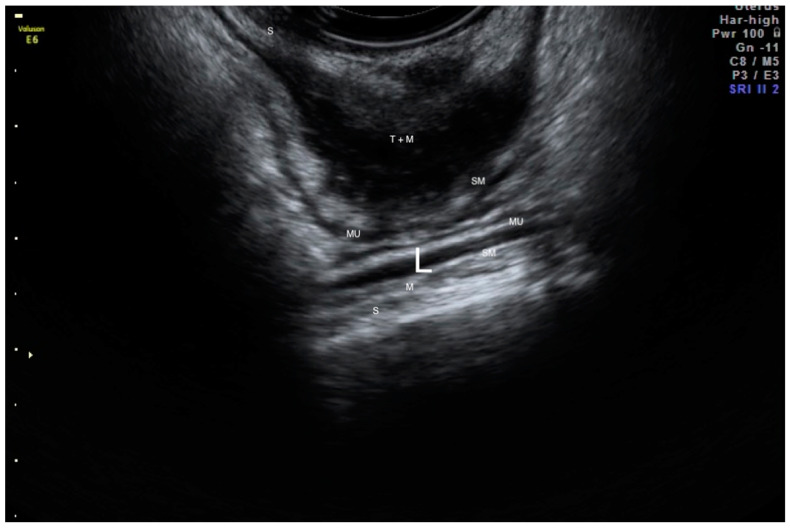
Big endometrial tumor of the rectum (T) which causes considerable occlusion of the bowel lumen (L). Layers of the intestine—serosa (S), muscle membrane (M), submucosa (SM), mucous membrane (MU).

**Figure 5:**
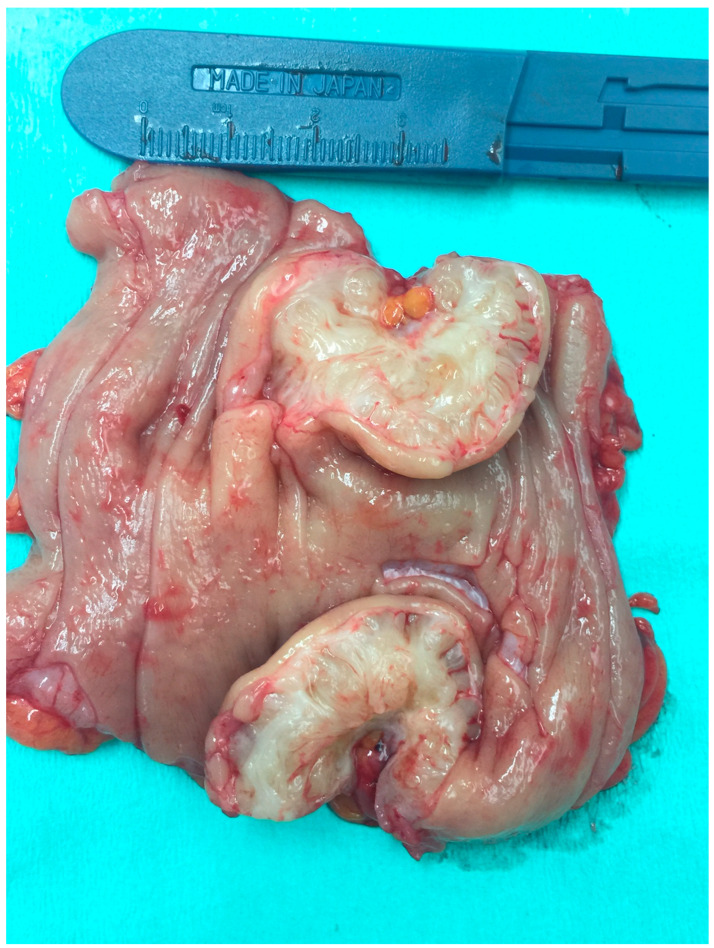
Histopathological material. Partially resected bowel due to deep bowel endometriosis.

The endometriotic lesion shown in Figure 2 and Figure 3, according to the #Enzian classification, falls into the P0, T0, O0, A0, B0, and C2 categories. Figure 3 shows that the method allows for estimation of the stage of bowel occlusion (at 30%). Thanks to the analysis of the measurement and stenosis of the bowel lumen we can evaluate the percentage of bowel lumen constriction. The intestinal lumen is noticeably (nearly 100%) constricted in Figure 4. The intestinal nodules were detected via RWC-TVS in 30 out of 31 examined patients. The mean dimension of the nodules measured via RWC-TVS was 23.61 mm (±9.4 mm), and the mean dimension of the histopathological samples was 26.03 mm (±18.5 mm). The difference between the dimensions obtained from RWC-TVS and histopathological examination was insignificant (*p* = 0.94). The results are shown in Table 2. Figure 5 presents a histopathological sample taken immediately after surgery that was later appropriately secured and handed over for further analysis. The greatest dimension of the nodule measured via RWC-TVS correlates with the size of the corresponding histopathological sample (R = 0.406, *p* = 0.03).

## 4. Discussion

Deep infiltrating endometriosis is a disorder that causes diagnostic issues and is contentious [32]. It causes a variety of uncomfortable symptoms, depending on where it is localized. The most common symptoms reported by the patients are dysmenorrhea, deep dyspareunia, acyclic pelvic pain, infertility, cyclic bowel, and urinary symptoms [16,17]. The intensity of these symptoms strongly correlates with the depth of the deep infiltrating endometriosis lesions and may reduce the quality of life of the affected women [33,34].

The development of diagnostic tools which will allow for assessment of deep infiltrating endometriosis is essential in selecting the best therapeutic approach. The selection of an appropriate diagnostic method allows for informed counseling and helps to select the appropriate surgical team and operation method and evaluate the expected complexity of the operation [26]. Clinical examination has limited use for confirming the expansion of the deep infiltrating endometriosis lesions, therefore it is crucial to use noninvasive imaging procedures before implementation of further treatment. Consequently, the time between the onset of the first symptoms and the successful clinical diagnosis of endometriosis can often be about 7–10 years [35]. There are two main diagnostic tools used in endometriosis diagnostics: ultrasonography and magnetic resonance imaging. Magnetic resonance imaging offers the possibility to obtain a complete pelvic evaluation with a single imaging procedure. However, it is not deprived of errors. The movements of the bowels may generate artifacts, which can result in overlooking or underestimating the size of endometriotic nodules in the bowel. As proved by Saba et al., performing the examination by an expert in endometriosis diagnostics improves the diagnostic accuracy in the preoperative assessment in comparison with a general radiologist [36,37]. Unfortunately, MRI is an expensive and long-lasting diagnostic procedure.

Because of that, MRI is not the first-line diagnostic tool but may be complementary to ultrasonography. While ultrasonography is considered as a “first-line” procedure not only due to its diagnostic usefulness, low cost, and rather small discomfort for the patient during examination, it can also be an operator-dependent and subjective technique. Ultrasound examinations are considered safe and are greatly acceptable by patients, as per acceptability studies in pregnancy and ovarian tumor screening populations [38]. The broad use of prenatal ultrasonography allows for the successful screening of pregnancy pathologies; however, doctors also detect a larger number of asymptomatic ovarian masses. Most of them are functional and benign. For instance, during screening examinations of ovaries, 11 to 41% of detected corpus luteum and simple cysts are wrongly classified as ovarian tumors. Ovarian masses that reach dimensions greater than 6 cm are indications for surgical treatment. It is recommended to perform an operation in the second trimester of pregnancy [39].

Di Giovanni et al. report in their study that an ultrasonographic method, such as transvaginal ultrasonography (TVS), has good sensitivity and high specificity for evaluating bowel endometriotic nodules when performed by a dedicated sonographer with extensive training and expertise in a specialized center. They also believe that transvaginal ultrasonography can be helpful with the decision-making in terms of the best surgical method for the patient [40]. Recently, a study reported that a sonographer who performed around 40 examinations could achieve proficiency [41]. In our study, all ultrasound evaluations were performed by experienced gynecologists trained in ultrasonography. Moreover, the study was performed in a reference center for the surgical treatment of endometriosis.

Over the last 10 years, ultrasound techniques and their implementation in clinical diagnosis have improved substantially. This has changed the quality of the noninvasive assessment of patients with suspected pelvic pathologies [42]. Taking different locations of endometriosis into consideration, the main challenge of diagnostic imaging is the detection of nonovarian disease [40]. An ultrasound scan allows us to visualize the presence of an irregular hypoechoic nodule, with or without hypoechoic or hyperechoic foci, and also allows us to assess the probable extension of the disease into pelvic structures [13,43]. Many different innovative ultrasound methods have been introduced in recent years, such as rectal water contrast transvaginal ultrasonography, vaginosonography, the ‘tenderness-guided’ approach, and the three-dimensional (3D) probes method [44]. The greater part of the published studies suggests that RWC-TVS has promising results for the preoperative detection of deep endometriosis infiltrating the rectosigmoid and may be easily implied in the diagnostic process [45]. This technique causes rather small discomfort to the patient as it is significantly less invasive than other techniques such as rectal endoscopic ultrasonography. On top of that, the patient can avoid general anesthesia and it is cheaper and more accessible than MRI [30]. However, RWC-TVS can be used only for detecting endometriotic lesions which are located below the rectosigmoid junction, because only nodules below this area are visible in ultrasonographic pictures [45].

In their study, Indrielle–Kelly et al. compare the sensitivity and specificity in detecting DIE by using two diagnostic methods—MRI and TVS. According to their research, the sensitivity and specificity for detecting DIE using both methods in the rectum are equal to 100%. Sensitivity for detecting DIE in rectosigmoid by MRI and TVS is 94%, whereas specificity is equivalent to 84% [46]. Barra et at. reported that RWC-TVS has a sensitivity equal to 95.2% and a specificity of 99.5% in detecting DIE when diagnosing rectosigmoid endometriosis [14]. Due to the higher availability and lower cost, ultrasonography seems to be the first-choice method for detecting DIE [47].

Valenzano Menada et al. verified that adding water contrast into the rectal lumen improves the accuracy of a deep infiltrating endometriosis diagnosis. In their study, it was shown that transvaginal ultrasonography combined with water contrast in the rectum is accurate in diagnosing rectal wall infiltration in women with rectovaginal endometriosis [45]. Fabio Barra et al. showed that transvaginal sonography and rectal water contrast transvaginal sonography were good diagnostic tools for the detection of deep infiltrating endometriosis. Both techniques detected endometrial changes in uterosacral ligaments, the vagina, and rectovaginal septum. However, RWC-TVS showed better performance in detecting rectosigmoid endometriosis. It allowed them to estimate the depth of the infiltration of endometriosis in the intestine wall and the distance between the nodules and anal verge. In their study, they showed that only RWC-TVS allows for the estimation of bowel lumen stenosis [14]. In our study, endometriotic nodules were detected in the intestines of all examined patients except for one. Unfortunately, because of the small study group in our study, due to the relatively rare occurrence of deep infiltrating endometriosis in the population, we were not able to confirm that adding RWC to TVS improves the accuracy of TVS alone during the diagnostic process. A study on a larger study group should be performed.

In Valenzano’s study, both the TVS and RWC-TVS results regarding the size of the lesions were in line with histological analysis performed after operational treatment [45]. In our study, the greatest dimension of the nodule measured via RWC-TVS also correlated with the corresponding size from the histopathological sample. The size measured using RWC-TVS was on average lower than the size seen in the histopathological results, but the difference was not statistically significant. Therefore, an assessment of the size of intestinal endometriotic nodules based solely on RWC-TVS may lead to underestimation. Further study on a bigger study group should be performed to investigate the accuracy of the assessment of the size of intestinal endometriotic nodules via RWC-TVS.

Ultrasonographic examination becomes permanently linked to gynecological surgical procedures. The gynecological surgeon sonologist can use ultrasound as an extension of the clinical examination. It provides rapid results and helps to assess the patient’s anatomy in real-time. MRI, on the other hand, allows for the evaluation of the anatomy on multiple planes, but it does not allow for dynamic visualization of the pelvic organs. According to Pascoal et al., MRI should be therefore considered a second-line technique in the diagnosis of deep infiltrating endometriosis [38].

Bowel endometriosis, because of non-specific symptoms, can be incorrectly diagnosed as a malignant tumor, inflammatory bowel disease, or other colorectal disorders. Hence, differential diagnosis of bowel endometriosis can be challenging. Not always imagining techniques can provide accurate recognition. Carvalho et al. describe a case report of a patient with non-specific colorectal symptoms. Because of problems with accurate diagnosis, they performed fine-needle aspiration (FNA) of the lesion. It was helpful and made the diagnosis possible [48]. Miwa et al. also present a case during which endoscopic ultrasound-guided fine needle aspiration (EUS-FNA) was useful to establish a diagnosis and determine the plan of further proceedings [49].

## 5. Conclusions

Deep bowel endometriosis is very challenging to diagnose and treat. Therefore, significant efforts in improving endometriosis detection tools, for example, the imaging methods, as well as a thorough evaluation of the patient’s symptoms, age, intention for postoperative hormonal treatment, and reproductive status are required to improve the confidence of clinical diagnosis. Extensive preoperative visual assessment of the diameter of the endometrial tumor, the stage of infiltration of the intestinal wall, and stenosis of the bowel lumen are helpful parameters for planning the relevant surgical treatment and team [17]. For the preoperative detection of deep bowel endometriosis, RWC-TVS is a very helpful approach. Although the technique has its limitations, it is often well tolerated by patients, has low cost, good accessibility, high specificity, as well as sensitivity in the detection of endometriotic rectosigmoid lesions. This diagnostic tool can help estimate the infiltration of the endometriotic lesion into the bowel wall, the distance from the anal verge, and the stage of stenosis of the bowel lumen [14]. Currently, transvaginal sonography and rectal water contrast transvaginal sonography are the first-choice methods for the preoperative diagnostic process. Surgeons, who frequently are also sonographers, can utilize these imagining techniques as helpful components of clinical examination. This technique allows for obtaining results in real-time, which can shorten the diagnostic process and initiate the treatment. Even though in our study the measurements of nodules obtained via RWC-TVS were marginally underestimated when compared with results from histopathological samples, the technique proves to be a highly useful tool in deep endometriosis diagnostics.

## Figures and Tables

**Table 1:** Incidence of endometriosis at different locations.

Localization of Endometriosis	Number of Patients	Percentage
Rectosigmoid	16	52%
Rectosigmoid + bladder	18	6%
Rectosigmoid + others	22	13%
Rectosigmoid + uterosacral ligaments and posterior vaginal fornix + others	23	3%
Rectosigmoid + uterosacral ligaments and posterior vaginal fornix + bladder	24	3%
Rectosigmoid + uterosacral ligaments and posterior vaginal fornix	30	19%
Unrecognized endometriosis	1	4%

**Table 2:** Descriptive statistics for the sizes of the endometriotic nodules obtained from ultrasound and histopathological examination. RWC-TVS—rectal water contrast transvaginal sonography, HPE—histopathological examination, M—mean, Min—minimal value, Max—maximal value, SD—standard deviation, *p*—probability in the Wilcoxon paired test.

	M	Min	Max	SD	*p*
RWC-TVS greatest size (mm)	23.613	0.00	45.00	9.42	0.94
HPE greatest size (mm)	26.033	8.00	90.00	18.49	

## Data Availability

All data generated or analyzed during this study are included in this published article.
